# Charged Molecules Modulate the Volume Exclusion Effects Exerted by Crowders on FtsZ Polymerization

**DOI:** 10.1371/journal.pone.0149060

**Published:** 2016-02-12

**Authors:** Begoña Monterroso, Belén Reija, Mercedes Jiménez, Silvia Zorrilla, Germán Rivas

**Affiliations:** 1 Centro de Investigaciones Biológicas, Consejo Superior de Investigaciones Científicas (CSIC), Madrid, Spain; 2 Instituto de Química-Física Rocasolano, Consejo Superior de Investigaciones Científicas (CSIC), Madrid, Spain; Laval University Cancer Research Centre, CANADA

## Abstract

We have studied the influence of protein crowders, either combined or individually, on the GTP-induced FtsZ cooperative assembly, crucial for the formation of the dynamic septal ring and, hence, for bacterial division. It was earlier demonstrated that high concentrations of inert polymers like Ficoll 70, used to mimic the crowded cellular interior, favor the assembly of FtsZ into bundles with slow depolymerization. We have found, by fluorescence anisotropy together with light scattering measurements, that the presence of protein crowders increases the tendency of FtsZ to polymerize at micromolar magnesium concentration, being the effect larger with ovomucoid, a negatively charged protein. Neutral polymers and a positively charged protein also diminished the critical concentration of assembly, the extent of the effect being compatible with that expected according to pure volume exclusion models. FtsZ polymerization was also observed to be strongly promoted by a negatively charged polymer, DNA, and by some unrelated polymers like PEGs at concentrations below the crowding regime. The influence of mixed crowders mimicking the heterogeneity of the intracellular environment on the tendency of FtsZ to assemble was also studied and nonadditive effects were found to prevail. Far from exactly reproducing the bacterial cytoplasm environment, this approach serves as a simplified model illustrating how its intrinsically crowded and heterogeneous nature may modulate FtsZ assembly into a functional Z-ring.

## Introduction

FtsZ, an essential protein required for division in most bacteria, forms a ring (Z-ring) at midcell thought to be responsible for initiating and driving cell constriction [1,2]. In the presence of GDP, FtsZ exhibits isodesmic oligomerization in a KCl and Mg^2+^ dependent manner [3]. The cooperative assembly of FtsZ into polymers, linked to its GTPase activity, has been extensively studied *in vitro*, especially for *Escherichia coli* FtsZ [2,4]. Addition of GTP triggers, above the critical concentration of polymerization (*Cc*) [1], FtsZ assembly into a narrow size distribution of polymers [5,6] whose final size and arrangement is highly variable depending on the solution conditions [2,5–7].

The importance of considering the crowded nature of the cytoplasm when studying the assembly of functional complexes *in vitro* has been largely acknowledged, since it determines key aspects for protein reactivity as available volume, preferential location or exclusion of a certain component, nonspecific attractive and repulsive interactions with background molecules or large structures, etc. Crowded conditions may modify the energetics of macromolecular reactions through excluded volume effects arising from the mutual impenetrability of macromolecules, providing a generalized nonspecific force that promotes macromolecular compaction and association [8]. The magnitude of this effect depends on the relative size of background and tracer molecules [8] as, for a given crowder size, the volume excluded decreases with the size of the tracer. Effects derived from volume exclusion can be compensated to a greater or lesser extent by additional specific and nonspecific interactions [9,10], highly system dependent as they vary with the chemical nature of the interacting species and the type of reactions studied. A reduced number of studies on mixed crowders, aimed at better mimicking the heterogeneous nature of the living systems, have been published. Reported studies on this type of crowded media cover in a qualitative or semiquantitative manner aspects as protein folding [11,12], protein stability [13] and effect on amyloid fibril formation [14]. Nonadditive effects of mixed crowders, reflected as an enhanced or reduced effect regarding the sum of those from the individual crowding agents, may have profound implications for macromolecular function inside cells [13].

The effect of macromolecular crowding on the noncooperative oligomerization of GDP-FtsZ has been previously studied in solutions containing BSA or hemoglobin as crowder agents [15]. In this study it was shown that crowders favor the formation of higher order oligomers of GDP-FtsZ and excluded volume models satisfactorily described the results obtained. The influence of macromolecular crowding on the GTP-FtsZ assembly has also been studied using Ficoll 70 or dextran, characterized by the promotion of bundles of FtsZ filaments at concentrations of these crowders above 100 g/L [16]. Under the millimolar magnesium conditions at which those experiments were conducted, the low critical concentration of polymerization of FtsZ hindered a precise evaluation of the effect of crowding on this parameter. More recently, the association of the highly flexible FtsZ filaments into higher order structures, very different depending on solution conditions, has been characterized principally by imaging methods [17]. To the best of our knowledge, there are no studies available in the literature dealing with the impact of proteins or combinations of crowding agents on GTP-FtsZ assembly.

Here, the influence of high concentrations of unrelated macromolecules including proteins on the GTP triggered FtsZ assembly was assessed attending specifically to critical concentration of polymerization, kinetics of depolymerization and arrangement into higher order structures. The micromolar magnesium conditions employed substantially increase the *Cc* value [6,18,19], allowing an appropriate determination of crowding effects, without altering FtsZ assembly behavior [19] and avoiding the unwanted unspecific oligomers promoted by crowders on the GDP-FtsZ form under higher Mg^2+^ conditions [15]. The evolution of the critical concentration of polymerization with the concentration of the crowders was compared with that expected for a pure volume exclusion behavior. The effects of the highly concentrated proteins were compared with those of inert and non-inert polymers and with those observed in mixtures of two crowder agents. In light of the results obtained the potential implications of the highly concentrated and heterogeneous cellular cytoplasm where the protein exerts its function on FtsZ assembly and hence on division are discussed.

## Materials and Methods

### Materials

Dextran T40 was from Amersham Pharmacia Biotech, Ficoll 70 was from General Electric, and PEG 20 and sucrose were from Merck. PEG 8, ribonuclease A (RNase A, 9.3 isoelectric point [20]) and salmon testes DNA were from Sigma. Ovomucoid (4.3 isoelectric point [21]) was from Worthington Chemicals. All crowders were used without further purification, equilibrated by extensive dialysis in the working buffer (50 mM Tris-HCl, pH 7.5, 500 mM KCl, 100 μM MgCl_2_). Final concentrations of the stock solutions were determined from the refractive index increment (dextran T40, 0.147 mL/g (APS Corp.); Ficoll 70, 0.141 mL/g [22]; PEG 20 and PEG 8, 0.136 mL/g [23,24]; and sucrose, 0.138 mL/g [25]) or spectrophotometrically (RNase A, ε_280_ = 9440 M^-1^ cm^-1^ (calculated from the sequence); ovomucoid, ε_280_ = 11275 M^-1^ cm^-1^ [26]). The water of the commercial 10 g/L DNA solution was evaporated using a SpeedVac and the pellet dissolved in the desired volume of working buffer to reach the final concentration.

### Protein purification and labeling

*E*. *coli* FtsZ was overproduced from *E*. *coli* BL21(DE3) transformed with the plasmid pMFV56 and purified by the Ca^2+^ induced precipitation method [3], stored at -80°C in the ionic exchange elution buffer and, immediately before use, dialyzed in the working buffer. Protein labeling with Alexa 488 carboxylic acid succinimidyl ester dye (Molecular Probes, Invitrogen) was performed in polymeric form as described [16,18]. The degree of labeling of FtsZ, estimated from the molar absorption coefficients of the fluorophore and the protein, was 0.3–0.5 moles of fluorophore per mole of protein.

### Steady-state fluorescence anisotropy

To determine the critical concentration of FtsZ polymerization, fluorescence anisotropy titrations of FtsZ-Alexa 488 were carried out using a PC1 photon counting steady-state ISS spectrofluorometer as detailed [18]. The concentration of FtsZ-Alexa 488 in the samples without crowders was usually 0.006 g/L (150 nM), being raised to 0.032 g/L under crowding conditions to improve the signal to noise ratio, as significant contribution of scattering was observed in some of the samples. Additional unlabeled FtsZ was added to achieve the final concentrations (typically between 0.02 and 1–1.5 g/L). Titrations were carried out in working buffer with the specified crowder at 20°C. 2 mM GTP was added to all samples. Isotherms correspond to the average of at least three independent experiments. The critical concentration of assembly was determined by fitting to the anisotropy isotherms the model described in [18], using user-written scripts and functions in MATLAB (Ver. 7.10, MathWorks, Natick, MA) and in Origin 7.0 with the same result.

To monitor the FtsZ depolymerization kinetics in the presence and absence of macromolecular crowders, the anisotropy of samples containing 1.5 g/L FtsZ was recorded with time at 20°C after adding 2 mM GTP.

### Light scattering

90° light scattering was recorded in a PC1 photon counting steady-state spectrofluorometer (ISS) at 350 nm and 20°C in working buffer, with and without crowder, using 3x3 mm cuvettes. Data at each concentration represent the average of at least three independent measurements after incubation of FtsZ with GTP ensuring stable signal. The change in scattering upon addition of GTP was measured at different FtsZ concentrations (typically between 0.02 and 1–1.5 g/L) and FtsZ critical concentration of polymerization was determined by fitting a linear regression model to the data points falling within the association regime as described in [16].

### Simulations based on volume exclusion theory

Simulations were done according to a simple model describing the dependence of the solubility of unassembled FtsZ (i.e. the *Cc*) with crowder concentration using user-written scripts and functions in MATLAB. The model assumes that FtsZ polymerization behaves as a first-order phase transition in which isolated molecules of FtsZ coexist at equilibrium with a condensed phase (the polymers):

$$Cc=\frac{{Cc}_{0}-C_{lim}}{\text{exp}\left(\text{ln}\;\gamma \right)}+\;C_{lim}$$

*Cc*_*0*_ is the FtsZ critical concentration of polymerization in dilute solution (0.56 g/L in our working buffer), *C*_*lim*_ is the amount of protein that remains unassembled (0 for all crowders but ovomucoid, 0.05 g/L as experimentally determined) and γ is the activity coefficient. The latter is defined in terms of the exclusion volume, concentration and masses of all species present in the solution [27]. The parameters used for the simulations were the effective volumes specified in the figure legends and the masses. The model considers crowders and proteins, including FtsZ, as spheres the size of which are determined by the nature of the interactions with the surrounding molecules. We assumed compliance with excluded volume behavior when the curve generated using the partial specific volumes of the species (or a close value) as exclusion volumes [28] was compatible with the experimental data, as the exclusion volume is a measure of solute-solute interaction [29]. Deviation from exclusion volume behavior was only assumed when the exclusion volumes required to describe the experimental data were substantially different from the partial specific volumes.

### Electron microscopy (EM)

Negative staining of FtsZ polymers was conducted as follows. GTP was added to a 1 g/L FtsZ stock solution, equilibrated in working buffer, to get a final concentration of 1 mM. After 1 min, solutions were adsorbed to carbon-coated grids and, 1 min later, stained for 1 min with 2% uranyl acetate. When in the presence of protein crowders, FtsZ stock concentration was reduced to the values specified in the figure captions in order to facilitate the visualization, provided the decrease did not modify the type of structure. Controls with no GTP were similarly prepared. Images were recorded on a TemCam-F416 CMOS camera (TVIPS) under low dose conditions at 50,000 × or 60,000 × nominal magnifications, on a JEOL-1200 electron microscope operated at 90 kV.

## Results

### Negatively charged crowders have a strong impact on FtsZ assembly

**Fig 1:**
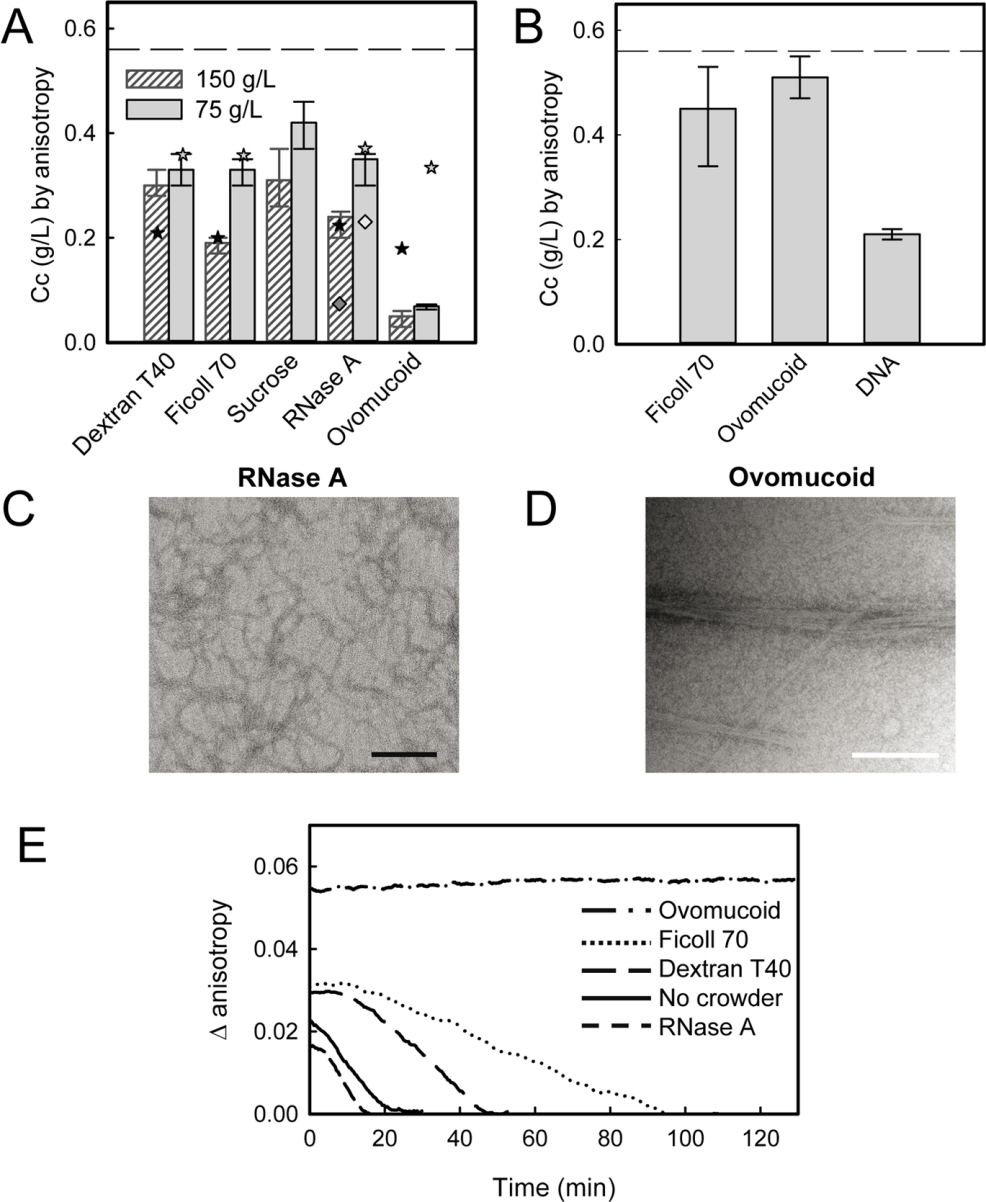
Effect of single crowders on FtsZ polymerization. (A) Effect of the specified crowders on FtsZ *Cc* of polymerization. Bars correspond to the average ± SD of the values determined for each specific crowder at that particular concentration (Table 1). The dashed line corresponds to the *Cc* value in the absence of crowder. Stars depict the theoretical *Cc* values for a pure volume exclusion behavior calculated for each crowder using their partial specific volumes as exclusion volume: υ_Dex_ = 0.61 mL/g [31], υ_RNase_ = 0.703 mL/g [32], υ_Ovo_ = 0.69 mL/g [33], and considering tetramers in the particular case of RNase A (calculated value for RNase monomers is shown as a diamond). Ficoll simulated data were calculated with an exclusion volume of 0.96 mL/g. υ_FtsZ_ = 0.74 mL/g [3]. (B) *Cc* values ± SD determined with 10 g/L Ficoll or ovomucoid and 11.8 g/L DNA. Dashed line as in A. (C) and (D) Structures induced on GTP-FtsZ polymers by the presence of RNase A (0.5 g/L FtsZ) and ovomucoid (0.25 g/L FtsZ), respectively. Crowder concentration was 150 g/L. Bar 200 nm. (E) Effect of different crowders (150 g/L) on kinetics of depolymerization of FtsZ (1.5 g/L). Profile in the absence of crowder is shown for comparison.

To study the influence of macromolecular crowding on FtsZ assembly, we analyzed the polymerization of FtsZ in solutions containing high concentrations of unrelated proteins or inert polymers. Fluorescence anisotropy and 90° light scattering measurements on samples containing 75 g/L RNase A or ovomucoid indicated that these proteins reduce the critical concentration of assembly of FtsZ with respect to that determined under the same conditions in dilute solution, being the reduction more dramatic for ovomucoid (Fig 1A and Table 1). Noteworthy, a higher basal scattering signal in the samples containing RNase A, probably arising from its tendency to self-associate [30] together with the fact that, in RNase A, FtsZ forms single stranded protofilaments yielding lower signal than bundles (Fig 1C), impaired the characterization of these solutions by scattering. A decrease of the *Cc* was also observed at the same concentration of the inert polymers Ficoll 70 and dextran T40 and of the osmolyte sucrose, under our experimental conditions (Fig 1A and Table 1). An increase of the crowders concentration to 150 g/L resulted in further reduction of the *Cc* value, indicating that the enhancement of FtsZ polymerization was exerted in a concentration dependent manner, although the difference in the case of ovomucoid was subtler. At both crowders concentrations, the *Cc* values determined in dextran T40 and Ficoll 70 solutions were comparable to those obtained for RNase A and much higher than the ones retrieved in the presence of ovomucoid. Sucrose had a lower influence that might result from its substantially smaller size (only slightly larger than the solvent) relative to that of FtsZ, attenuating its crowding effect. For the other crowders, however, the size does not account for the tendency observed in the *Cc*. In particular, it does not explain the pronounced effect of ovomucoid.

**Table 1:** Critical concentration of polymerization of FtsZ in the absence and presence of different crowders.

	Critical concentration of polymerization (g/L)	
Crowder/concentration	Anisotropy	90° Scattering
---	0.56 (+0.06/-0.02)	0.60 ± 0.10
Ficoll 70		
75 g/L	0.33 (+0.02/-0.03)	0.2 ± 0.1
150 g/L	0.19 (+0.01/-0.02)	0.20 ± 0.02
Dextran T40		
75 g/L	0.33 ± 0.03	0.31 ± 0.07
150 g/L	0.30 (+0.03/-0.02)	0.3 ± 0.1
Sucrose		
75 g/L	0.42 (+0.04/-0.05)	0.45 ± 0.01
150 g/L	0.31 (+0.06/-0.05)	0.29 ± 0.06
Ribonuclease A		
75 g/L	0.35 (+0.01/-0.05)	---
150 g/L	0.24 (+0.01/-0.04)	---
Ovomucoid		
75 g/L	0.069 (+0.004/-0.006)	0.08 ± 0.02
150 g/L	0.05 (+0.01/-0.02)	0.08 ± 0.01

*Cc* determined by fluorescence anisotropy and 90° scattering. In 50 mM Tris-HCl, pH 7.5, 500 mM KCl, 100 μM MgCl_2_ buffer.

Among the crowders tested, ovomucoid is the only one negatively charged under our working conditions, as it is FtsZ (isoelectric point 4.9 [34]). The large effect observed for this protein compared with the non-charged polymers or RNase A, a positively charged protein, prompted us to test the effect of other negatively charged species such as DNA on FtsZ polymerization. The decrease in the *Cc* value at ∼10 g/L DNA was comparable to that obtained in the presence of 25 g/L ovomucoid (0.21 ± 0.01 and 0.18 ± 0.05 g/L, respectively) and considerably larger than that with 10 g/L of this protein or Ficoll 70 (Fig 1B). The high viscosity of the DNA solutions precluded the performance of *Cc* measurements at higher concentrations. Therefore, we found that the negatively charged macromolecules, ovomucoid and DNA, produced large effects on FtsZ polymerization compared with neutral or positively charged crowders, likely due to significant electrostatic repulsion between the crowder molecules themselves [35] and with FtsZ that carries negative charge as well.

The structures of FtsZ filaments appearing in EM images at 150 g/L of either of the two crowder proteins, RNase A and ovomucoid, were different (Fig 1C and 1D). Single stranded filaments were found in the case of RNase A, while the structures observed in the presence of ovomucoid consisted of bundles, apparently thinner than those induced by Ficoll or dextran [16]. Single stranded filaments were also observed at 150 g/L sucrose and at ∼10 g/L DNA. Control experiments indicated that crowders by themselves were not able to promote polymerization of GDP-FtsZ, and that the GTP-FtsZ structures observed under our experimental conditions were independent of Mg^2+^ concentration in the 0.1–5 mM range.

The disassembly of the polymers was different in the solutions containing high concentrations of the two crowder proteins, probably related with the different arrangement they present (Fig 1E). Thus, in RNase A solutions the lifetime of the FtsZ structures was comparable to that of the polymer in dilute solution. In contrast, the stability of the bundles in the presence of ovomucoid was such that depolymerization was not observed within the measurement interval. The lifetime of the bundles induced by Ficoll 70 or dextran was intermediate between those found in the solutions of the two protein crowders. The different kinetics of disassembly of the bundles obtained with ovomucoid and Ficoll or dextran suggests that the actual arrangement of the protofilaments into bundles is different in each case.

### Negatively charged crowders exert an effect beyond volume exclusion on FtsZ assembly

**Fig 2:**
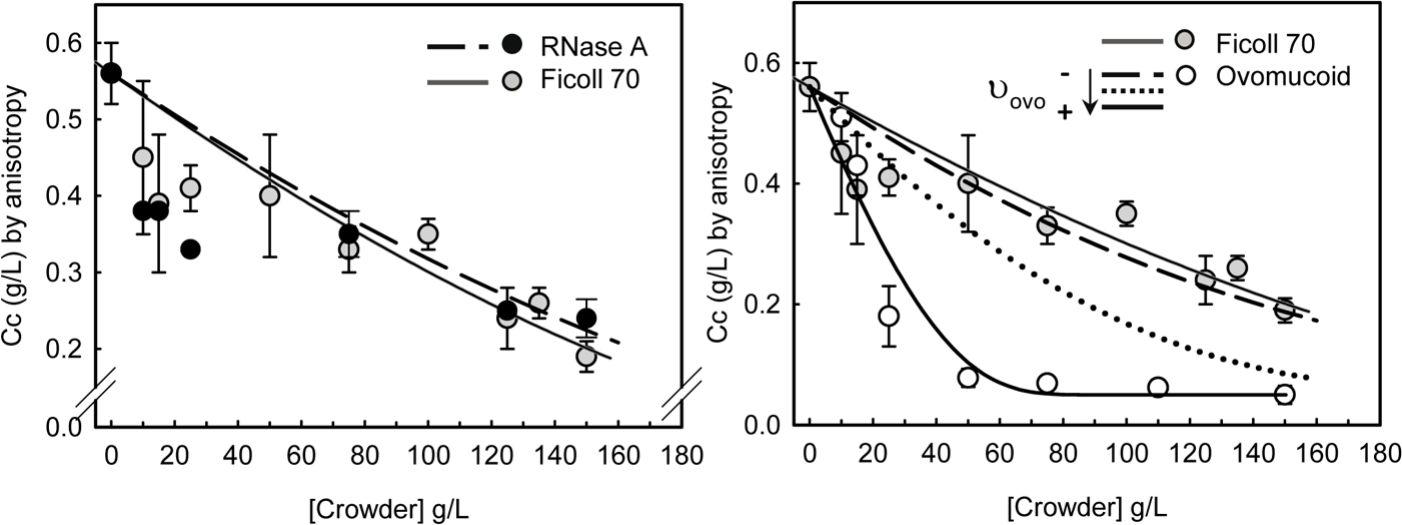
Concentration dependent effect of Ficoll 70, ovomucoid and RNase A on the polymerization of FtsZ. Symbols correspond to the variation of FtsZ *Cc* with the crowders concentration ± SD, average of at least three independent measurements. Lines correspond to the simulations according to the volume exclusion model detailed in the main text. υ_FtsZ_ = 0.74 mL/g [3], υ_Ficoll_ = 0.96 mL/g, υ_RNase_ = 0.703 mL/g [32] (tetramers). For ovomucoid, υ_Ovo_ = 0.69 mL/g (dashed black, pure volume exclusion behavior), υ_Ovo_ = 1.61 mL/g (dotted black, repulsion with like molecules [36]) and floating the parameter to υ_Ovo_ = 6.6 mL/g (solid black, accounting for additional effects). Arrow in the legend indicates the sense in which the exclusion volume increases.

To get further insight into the effect of the crowder proteins on the assembly of FtsZ, the evolution of the *Cc* with the concentration of the proteins and of Ficoll 70 was studied. The *Cc* of polymerization of FtsZ was found to decrease as the concentration of the individual crowders was increased in the solution (Fig 2). This decrease was more pronounced for ovomucoid, that largely reduced the *Cc* when at concentrations up to 50 g/L, value above which the *Cc* seemed to reach a plateau. The trend observed with the concentration of RNase A, however, was similar to that found for Ficoll 70, which gradually increased FtsZ assembly with the concentration of crowder, as expected for an effect purely due to excluded volume (see below).

Assuming that the assembly of FtsZ behaves as a first-order transition in which isolated molecules coexist at equilibrium with the polymers, a semiquantitative estimate of excluded volume effects on FtsZ polymerization can be obtained according to a simple model describing the dependence of the *Cc* with crowder concentration, determined by the activity coefficient that takes into account the exclusion volume, concentration and masses of all species present [27]. The experimentally determined values for the *Cc* in Ficoll were compatible with simulations according to this model (Figs 1A and 2) with an exclusion volume fairly close to its partial specific volume, 0.68 mL/g [37]. It should be considered that the exclusion volume does not necessarily equal the partial specific volume, as big polymers can deviate from the spherical shape and, therefore, modelling values are only approximate. Equivalent simulations for dextran at the two concentrations at which the *Cc* was determined showed a good agreement with the experimental data as well (Fig 1A), indicating that the enhancement of the tendency of FtsZ to polymerize produced by these inert crowder polymers is essentially that expected for a pure excluded volume effect.

For RNase A, the experimentally observed reduction in the *Cc* was smaller than expected from calculations using the excluded volume model, assuming that RNase A behaves as a monomer (Fig 1A). This may be explained by the tendency of RNase A to self-assemble and, indeed, the experimental data are compatible with simulations considering trimers or tetramers [30] of RNase A (Figs 1A and 2), which suggests that this protein crowder influences FtsZ assembly principally via steric hindrance. The case of ovomucoid clearly separated from this behavior though, and simulations using an exclusion volume equal to its partial specific volume or even a larger value that takes into account the occurrence of repulsive interactions between ovomucoid molecules at high concentrations (1.61 mL/g [36]), rendered values clearly underestimating the experimentally determined effect, accounted for by a further increase of this parameter (Figs 1A and 2). This strongly suggests that additional interactions, likely electrostatic repulsion between FtsZ and ovomucoid, further enhance the effect on the *Cc* derived from the repulsion of ovomucoid with like molecules [35].

### Nonadditive effects on FtsZ assembly prevail in mixtures of crowding agents

**Fig 3:**
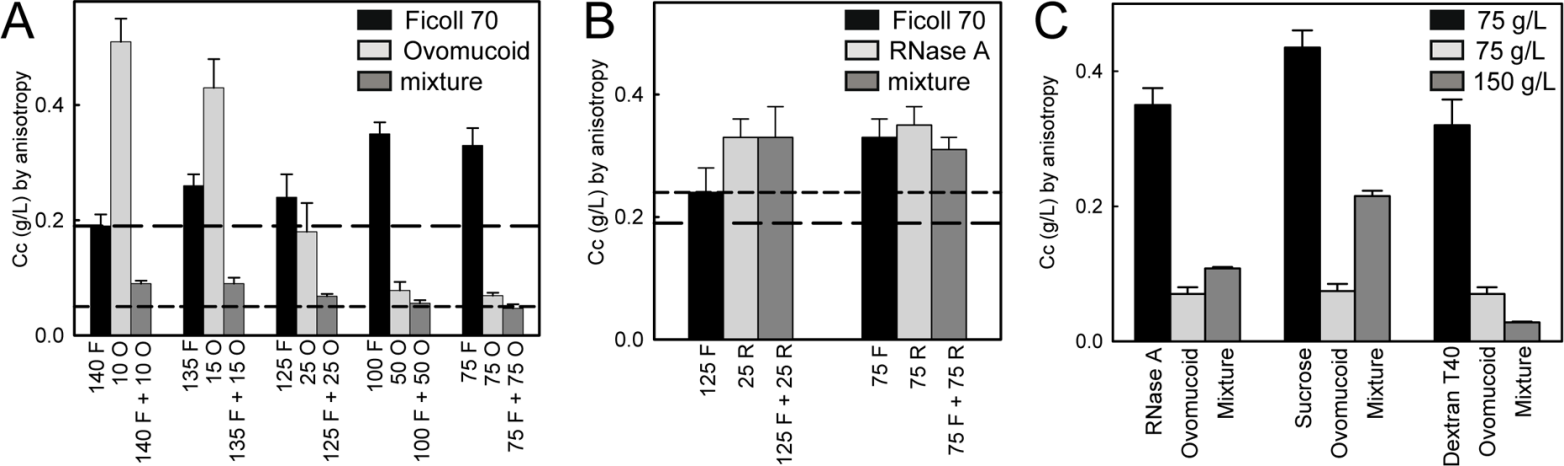
Effect of mixtures involving protein crowders on the polymerization of FtsZ. (A) and (B) *Cc* values determined in the presence of the specified crowders and their mixtures up to a total crowder concentration of 150 g/L (whole set of values in Table 2). In the x axis, F, O and R are Ficoll 70, ovomucoid and RNase A, respectively, and the numbers correspond to their concentration alone or in the mixtures, in g/L. Long and short dashed lines correspond to the *Cc* values in the presence of 150 g/L Ficoll (A and B) and ovomucoid (A) or RNase A (B), respectively. (C) *Cc* of FtsZ assembly in the presence of the individual crowders specified in the figure and their mixtures (50%). Values correspond to the average ± SD of at least three independent measurements.

The behavior of FtsZ regarding polymerization in solutions containing mixtures of crowding agents was first evaluated by assessment of the influence of the inert polymer typically used in crowding studies, Ficoll 70, on the effects observed for the two protein crowders studied, RNase A and ovomucoid. Determinations of the *Cc* for mixtures containing different ratios of Ficoll 70 and ovomucoid, at a final 150 g/L concentration, showed that the *Cc* for the mixtures was always lower than for the individual crowders at their concentration in the mixture and even for 150 g/L Ficoll (Fig 3A and Table 2). At ovomucoid: Ficoll ratios higher than 1:1, the effect on the *Cc* is mainly driven by the protein crowder and is saturated as no significant variation within this ovomucoid concentration interval was found. The presence of Ficoll above 75 g/L substantially enhanced the reduction of the *Cc* regarding that in the presence of ovomucoid at its concentration in the mixture, bringing the *Cc* to values close to those obtained at the 150 g/L ovomucoid limit. This blatant effect was more clearly observed with ovomucoid concentrations as low as 5–15 g/L, at which this protein crowder by itself only produced a moderate decrease in the *Cc* (Table 2 and Fig 3A). This seems to indicate that the joint interaction between FtsZ and the two additives in the mixture is nonadditive, as depending on the crowders ratio the trend of *Cc* values varies and does not correspond to a simple sum of the effects of the two individual additives. The dependence of *Cc*, therefore, might be determined by the concourse of additional nonspecific interactions, presumably electrostatic repulsion due to the negative charges between ovomucoid and FtsZ under our working conditions, which are already present with pure ovomucoid and potentiated by Ficoll. The heterogeneity of the structures observed already with each single crowder, set aside that under mixed crowding conditions, precluded further quantitative characterization of this complex system that was here studied only semiquantitatively. Similar behavior was found for the mixture of ovomucoid with dextran, in a 1:1 ratio up to 150 g/L, that resulted in a *Cc* smaller than in the presence of either of the crowders alone at the concentration in the mixture and even than with 150 g/L dextran, being close to that obtained at 150 g/L ovomucoid (Fig 3C). This suggests that, when mixed with ovomucoid, inert polymers do not counteract but potentiate the effects of the crowder protein.

**Table 2:** Critical concentration of polymerization of FtsZ in the presence of mixtures of Ficoll 70 and ovomucoid in different proportions.

Concentration in the mixture (g/L)		Critical concentration of polymerization (g/L)	
Ficoll 70	Ovomucoid	Anisotropy	90° Scattering
150	---	0.19 (+0.01/-0.02)	0.20 ± 0.02
145	5	0.12 ± 0.02	0.14 ± 0.05
140	10	0.09 ± 0.01	0.07 ± 0.03
135	15	0.09 ± 0.01	0.05 ± 0.01
125	25	0.068 ± 0.004	0.06 ± 0.03
100	50	0.056 (+0.006/-0.004)	---
75	75	0.047 (+0.005/-0.009)	0.05 ± 0.01
50	100	0.041 (+0.005/-0.002)	---
---	150	0.05 (+0.01/-0.02)	0.08 ± 0.01

Total crowder concentration was 150 g/L. *Cc* measured by fluorescence anisotropy and 90° scattering. In 50 mM Tris-HCl, pH 7.5, 500 mM KCl, 100 μM MgCl_2_ buffer.

Determinations of the *Cc* in mixtures containing RNase A and Ficoll at a final total 150 g/L concentration showed a very similar behavior at the two different ratios tested (1:1 and 1:5) (Fig 3B). The *Cc* for these mixtures was higher or equal than for the individual crowders at their concentration in the mixture and for the single crowders at 150 g/L. Given that, when used individually, both crowders had comparable effects on the *Cc* of FtsZ and that data can be adequately explained by a pure exclusion volume behavior, the observed effect may perhaps be explained by a further enhancement of the tendency of RNase A to self-associate by Ficoll, which would result in lower excluded volume. The behavior found for the mixtures of RNase A and Ficoll was then remarkably different from that observed for the mixtures of ovomucoid with the inert polymers.

FtsZ polymerization was also studied in solutions containing mixtures of the two crowder proteins. Measurements of the *Cc* in the simultaneous presence of RNase A and ovomucoid (1:1) showed values in between those of each crowder at their concentration in the mixture (Fig 3C). This result may be explained by the positive charge of RNase A at the working pH counteracting effects derived from the negative charge of ovomucoid, reinforcing the idea that the large influence observed for ovomucoid was likely related to an electrostatic phenomenon. Alternatively, a smaller effect on FtsZ for this mixture could be expected in the event of interaction between ovomucoid and RNase A that would result in fewer, larger crowder particles instead of more, smaller crowders [29].

Another additive we found to counteract the deep enhancement of FtsZ assembly provoked by ovomucoid was sucrose. Thus, the *Cc* value determined in 1:1 mixtures of ovomucoid and sucrose, total 150 g/L concentration, lay again intermediate between the values measured for each crowder at 75 g/L (Fig 3C). The purely steric excluded volume behavior of this mixture [35] allows discarding any unspecific interaction between both crowders hampering their effect on FtsZ. The stabilizing effect on proteins exerted by sucrose has been attributed to preferential hydration, or exclusion of sucrose nearby the protein surface, minimizing the surface accessible to solvent [38]. Counteraction of the ovomucoid effect on FtsZ when mixed with sucrose might arise, therefore, from the reduction in the area of FtsZ exposed to the protein crowder and/or a decrease in the occurrence of charge repulsion interactions because of hydration.

### PEG strongly enhances the tendency of FtsZ to polymerize at concentrations below the crowding regime

PEG, widely used in crowding and phase separation studies, has been profusely described to have noticeable effects on the thermodynamic activity of actin (see, for instance, [39–41]), a protein that shares with FtsZ the presence of a nucleotide and a bivalent cation bound to the monomer and the ability to polymerize above a critical concentration [41]. Although allegedly used as a crowder agent, concentrations employed in some of these studies were well below those considered to yield crowding conditions.

**Fig 4:**
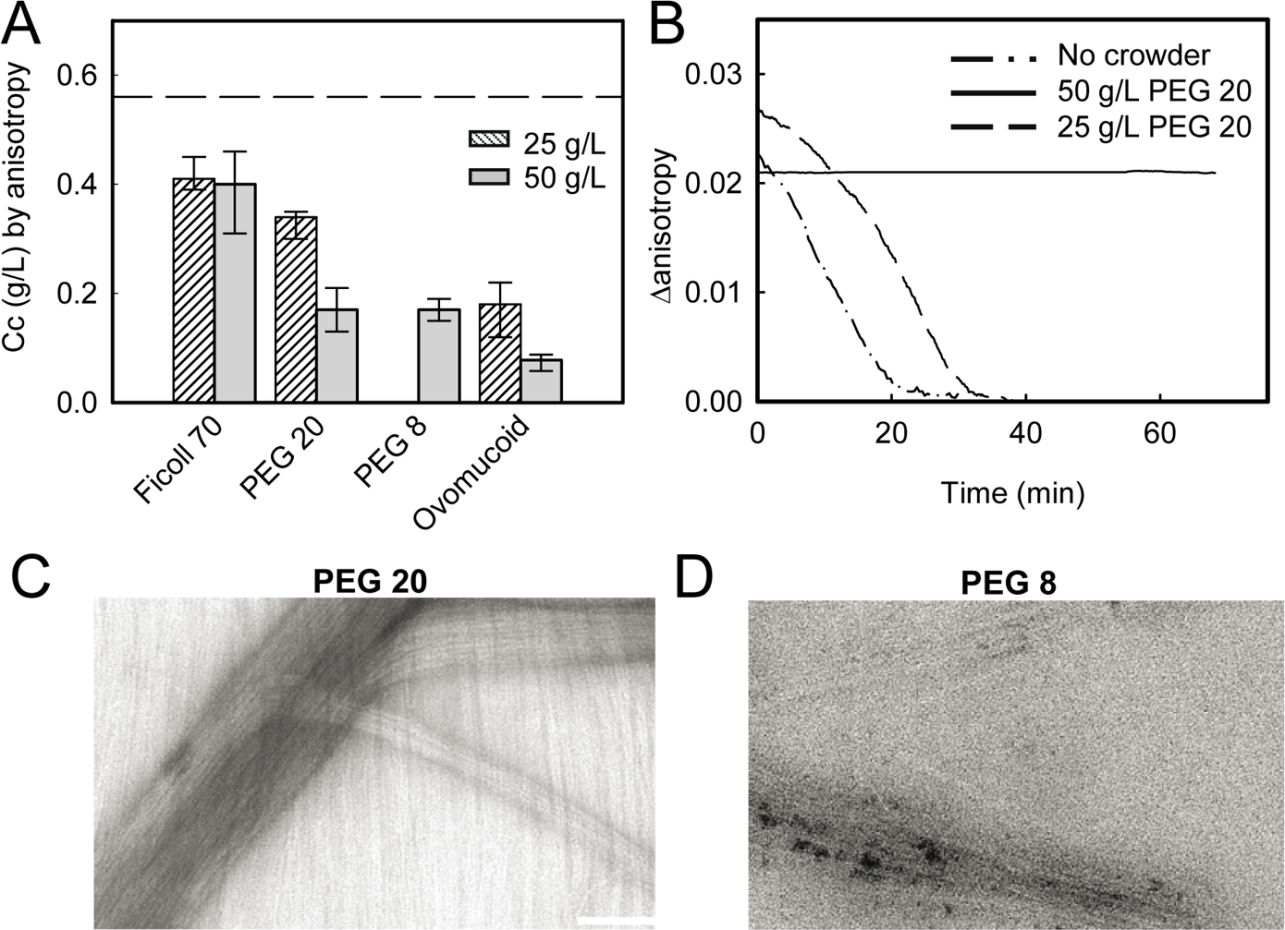
Effect of non-inert polymers on FtsZ polymerization. (A) *Cc* values ± SD determined with the specified crowders. Dashed line depicts the *Cc* value in the absence of crowder. (B) Effect on the depolymerization kinetics of FtsZ (1.5 g/L) of PEG 20. Profile in the absence of crowder is shown for reference. (C) and (D) EM images of GTP-FtsZ polymers in the presence of PEG 20 and PEG 8, respectively. FtsZ concentration was 1 g/L, and crowder concentrations were 50 g/L. Bar is 90 nm.

Measurements of the *Cc* of assembly of FtsZ in the presence of PEG 20 or PEG 8 showed that this kind of polymers also decreased the value with respect to that found in dilute solution, being the effect greatly strengthened at 50 g/L (Fig 4A). Visible aggregation of the samples limited the characterization of FtsZ polymers at higher concentrations of PEG.

The structures of FtsZ polymers generated by these additives were variable, ranging from discrete arrangements of grouped filaments (PEG 8) to thick bundles (PEG 20; Fig 4C and 4D). It is worth to mention that, among the crowders here tested, PEGs were the only ones inducing different structures depending on FtsZ concentration, as at 0.25 g/L protein the single stranded filaments formed in dilute solution upon GTP addition remained so in the presence of these additives. In the presence of 50 g/L PEG 20 (as well as with PEG 8), depolymerization did not occur in the time interval tested (Fig 4B), as also observed with 150 g/L ovomucoid. This may indicate a similar arrangement of the protofilaments to form bundles in the presence of the two kinds of crowders. Slow depolymerization dynamics has been previously reported also for FtsZ bundles formed in the presence of high concentration of secondary cations [2].

## Discussion

In this work we show that crowding conditions due to high concentrations of unrelated proteins, alone or mixed with other crowders, result in an enhancement of the tendency of FtsZ to form polymers reflected in a decrease of the critical concentration of polymerization. This effect is substantially more pronounced in the presence of ovomucoid compared with RNase A, which renders effects of magnitude similar to those by inert polymers like Ficoll and dextran, which act essentially via excluded volume [42], and osmolytes like sucrose. The large increase in the exclusion volume of ovomucoid regarding that expected for a protein of its size strongly suggests that its influence on FtsZ polymers is mediated by additional factors. One of these factors is likely the electrostatic repulsion between the negative charges of FtsZ and ovomucoid at the working pH. Experimental evidences point towards this direction, namely the counteracting behavior of the positively charged protein RNase A, but not of uncharged polymers, when mixed with ovomucoid and the strong promotion of FtsZ assembly obtained in the presence of DNA. Another type of crowders we found to have strong impact on FtsZ polymerization even at low concentrations are PEGs. These polymers are uncharged, and hence their large influence on FtsZ assembly is not related with electrostatic effects. Instead, PEG molecules have been shown to occupy most of the aqueous volume, excluding a small fraction into which large molecules are crowded [43], thus exerting a large and predominant repulsive effect. Accordingly, PEGs tend to induce macromolecular association and compaction [44] beyond that predicted by models purely based on volume exclusion [45,46], as we found here for FtsZ polymerization.

According to our results, protein crowders like RNase A at 150 g/L do not promote bundling of FtsZ polymers, in contrast to that previously described for the inert polymers Ficoll and dextran above 100 g/L [16] and that observed here for PEG at even lower concentrations. Previous studies on actin filaments using unrelated macromolecules like PEG or proteins not directly binding to them [40,47] showed that higher protein concentrations (>150 g/L in the case of ovalbumin [47]) are required to induce bundling, as might be the case here with ribonuclease. In contrast, it is possible that the additional effects exerted by ovomucoid would be the cause of the arrangement into structures thicker and longer than those expected to be induced by a crowder protein, substantially more stable in time than single stranded filaments.

As with individual crowders, the mixtures also enhanced the polymerization of FtsZ with respect to that observed in dilute solution, irrespective of the crowders chosen. Among the mixtures involving proteins, nonadditive effects were more frequently found, at least for the combinations showing a clear behavior at a single ratio (ovomucoid with RNase A or sucrose) and for the ones we have examined in more detail (ovomucoid or RNase A with Ficoll). Deviation from additivity seems to arise from different factors including counteraction of electrostatic effects, oligomerization of one of the crowders in the presence of the other or even interaction between the two crowders. Nonadditive behavior has been predicted to have a large impact on reactions occurring in biological environments [11], which are heterogeneous by definition. The semiquantitative characterization of FtsZ polymerization in mixtures of crowding agents shown here adds up to the scarce studies on mixed macromolecular crowding, mainly centered on protein refolding rather than on protein association.

Our results show a remarkable impact of protein crowders with a prevalence of nonadditive effects on the structural and thermodynamic aspects of FtsZ assembly that may have a strong influence on Z-ring formation in a heterogeneous environment of complex composition containing proteins, as it is the cytoplasm. In this sense our results, even obtained under simplified conditions using binary mixtures, illustrate how the excluded volume effects exerted on the same system are tuned in either sense (enhancement or reversion) by the presence of charged crowders. It must be taken into account that this behavior is determined by all elements involved, so the charge of the molecules studied under that particular conditions is also determining the overall effect observed, as well as the presence of other elements that participate in the reactivity of the systems as boundaries and confinement.
